# HtrA1 as a promising tissue marker in cancer: a meta-analysis

**DOI:** 10.1186/s12885-018-4041-2

**Published:** 2018-02-06

**Authors:** Emma Altobelli, Paolo Matteo Angeletti, Manrico Morroni, Valerio Filippo Profeta

**Affiliations:** 1 Department of Life, Health and Environmental Sciences, University of L’Aquila, L’Aquila, Italy; 2 Head of Epidemiology and Biostatistics Unit, ASL Teramo, Teramo, Italy; 3 Department of Life, Health and Environmental Sciences, University of L’Aquila, L’Aquila, Italy; 4 Department of Experimental and Clinical Medicine, Section of Neuroscience and Cell Biology, School of Medicine, Università Politecnica delle Marche, Ancona, Italy; 5 Department of Community Health, Teramo, Italy

**Keywords:** HtrA1, Cancer, Meta-analysis

## Abstract

**Background:**

HtrA1 is expressed in a variety of normal human tissues and seems to be involved in numerous physiological processes as well as tumorigenesis. This study reports the results of a meta-analysis that was performed: to compare HtrA1 expression as mRNA and protein, in cancer tissue versus non-cancer tissue and to assess overall survival in relation to low or medium-high HtrA1 tissue expression.

**Methods:**

The PRISMA method was used for study selection. OR and HR with 95% confidence interval was used as a measure of effect size as appropriate. A random-effects model was applied to account for different sources of variation among studies. Heterogeneity across studies was assessed using Q statistic. Sensitivity analysis was conducted to check the stability of study findings. Egger’s regression method was applied to test funnel plot asymmetry.

**Results:**

Sensitivity analysis indicated the stability of meta-analytic findings in each meta-analysis. The study found a significantly different HtrA1 expression in cancer and non-cancer tissue. The meta-analysis of the prognostic studies showed a different survival according to HtrA1 expression.

**Conclusions:**

The present data may provide a contribution to future work directed at exploring the role of HtrA1 in tumor development and progression and at establishing whether it may be used as a promising tissue marker for some tumors.

**Electronic supplementary material:**

The online version of this article (10.1186/s12885-018-4041-2) contains supplementary material, which is available to authorized users.

## Background

The members of the widely conserved high-temperature requirement A (HtrA) family of homo-oligomeric serine proteases are involved in a number of mammalian cellular processes that include growth [1], maintenance of mitochondrial homeostasis [2], apoptosis [3], and protein quality control [4]. HtrA1 has been reported to regulate such processes through modulation of growth factor systems, like the system mediated by the extracellular protein transforming growth factor β (TGF-β) [5] as demonstrated by the association of its expression with specific tumor behaviors.

Mammalian HtrA1 is connected to tumorigenesis [6, 7]; in particular, it is down-regulated in ovarian [8], thyroid [9], endometrial [10], and breast cancer [11], in hepatocellular carcinoma [12], and colorectal cancer [13] while its up-regulation seems to induce suppression of tumor cell proliferation and migration in highly invasive melanoma [6]. Indeed, several lines of evidence indicate that HtrA1 functions as a tumor suppressor in various solid tumors, such as ovarian and lung cancer and mesothelioma [8, 14, 15]. It also participates in regulating cancer cell apoptosis, invasion, and metastasis processes [16]. Distant metastasis, the final stage of solid tumors, is involved in most cancer deaths [16].

By using different experimental techniques, Lorenzi and colleagues [17] have found that HtrA1 protein expression is downregulated in urothelial cancer tissue regardless of tumor grade and stage, and suggested that urinary HtrA1 protein may be used as an early and highly sensitive and specific biomarker for this neoplasm.

Finally, according to recent evidence HtrA1 downregulation induces the acquisition of phenotypes such as increased proliferation, delayed onset of senescence, altered centrosome number and positioning and polyploidy, all hallmarks of tumor cells [18].

In a previous review, our group explored the potential role of HtrA1 as a tumor marker and/or prognostic factor [19].

This study reports the results of a meta-analysis that was performed: i) to compare HtrA1 expression as protein and mRNA, in cancer tissue (C) versus non-cancer tissue, i.e. healthy control (HC) tissue and normal-looking (NL) tissue; and ii) to assess overall survival in relation to low or medium-high HtrA1 tissue expression in different cancers.

## Methods

Literature search. Relevant studies were identified as of June 2017 using the following databases: PubMed, Embase, Web of Science, Cochrane Library, Scopus, and clinical trial registers (clinicaltrial.gov, clinicaltrialsregister.eu). A manual search was also performed. There were no limitations as to the year of publication. Only studies in English were considered. The reference lists of all studies were screened by two independent reviewers (PMA and MM); any disagreements were resolved by a methodologist (EA). The following keys words were used: HtrA1 OR PRSS11 protein, human OR L56 protein, human OR protease, serine, 11 (IGF binding) protein, human OR high-temperature requirement factor A1, human OR HtrA serine peptidase 1, human AND Neoplasm OR Tumors OR Tumor OR Neoplasia OR Cancer OR Cancers. Only studies assessing HtrA1 (mRNA or protein) as a diagnostic or prognostic tumor marker were considered. Studies were selected using the PRISMA statement (Additional file 1: Figure S1) [20]. The excluded studies and the PRISMA check list are reported in Additional file 2: Table S1 and Additional file 3: Table S2. The general characteristics of each study (tumor site, sample size, patient population) are reported in Table 1. The Newcastle-Ottawa was used for case-control studies was used (Additional file 4: Table S3).

**Table 1:** Characteristics of the studies included in the meta-analysis

TUMOR SITE	PATIENT POPULATION		HtrA1						RESULTS
	Cancer and Normal-looking	Healthy controls	Setting		Laboratory		Normalization		
			Tumor Marker	Prognostic Marker	Protein	mRNA	Housekeeping	Healthy tissues	
Colo-rectum [13]	*N* = 37 M = 22 F = 15 mean age ± sd 65.5 ± 9.4	*N* = 36 M = 13 F = 23 mean age ± sd 53.3 ± 10.0	✓		✓			✓	Differences between cancer and healthy control tissue *p* = 0.0001 Difference between cancer and normal-looking tissue *p* = 0.001
Liver [12]	*N* = 60 M = 25 F = 35, mean age 51.5	*–*	✓	–	–	✓	✓ GADPH		Difference between cancer and normal-looking tissue *p* < 0.0001
Stomach [34]	*N* = 42 M = 22 mean age ± sd 53.4 ± 9.9 F = 20 mean age ± sd 50.3 ± 14.1	*–*	✓	–	–	✓	✓ B-ACTIN		Normal gastric tissue, positive staining 83.3% Tumor tissue, negative staining 95.0%
Bladder [17]	*N* = 68, M = 50 F = 18 mean age ± sd 68.2 ± 7.0	*–*	✓	–	✓	✓	✓ B-ACTIN	✓	Difference between cancer and normal-looking tissue p < 0.0001 for protein. Difference for mRNA not significant *p* = 0.422
Breast [11]	*N* = 131 all females	–	–	✓		✓	✓ GADPH		Better survival in high expression OS HR 0.45 (0.23–0.90) *p* = 0.023
Esophagus [Yu] [35]	*N* = 63 M = 50 F = 10 mean age 73.4 range 45–79	–	✓	–	✓	✓	✓ GADPH B-ACTIN	✓	Difference between cancer and normal-looking tissue *p* < 0.05 as protein, *p* = 0.004 as mRNA
Esophagus [Xia] [36]	*N* = 115 Age < 60 *N* = 52 Age > 60 *N* = 63	–	✓	✓	✓	✓	✓ B-ACTIN		Difference between cancer and normal-looking tissue p < 0.05 both protein and mRNA
									Better survival in high expression OS: HR 0.75 (95 CI 0.38–1.4) *p* = 0.433^a^
Liver [37]	*N* = 50 M = 42 mean age ± sd 52.4 ± 9.9 F = 8 mean age ± sd 50.3 ± 14.1	–	✓	✓	✓	–	–	✓	Difference between cancer and normal-looking tissue *p* = 0.45
									Better survival in high expression: OS: HR 0.59 (95 CI 0.14–2.37) *p* = 0.486^a^
Stomach [38]	*N* = 80 M = 51, F = 29 mean age, 64 range, 32–82	–		✓	✓			✓	OS: HR 0.55 (95% CI 0.32–0.96) *p* = 0.037
Thyroid [9]	*N* = 20 age not reported		✓	✓	✓			✓ GADPH	Not statistically significant
Endometrium [Mullany] [10]	*N* = 184 mean age ± sd 66.1 ± 11.3	–		✓	✓		✓ B-ACTIN		OS HR: 0.29 (95% CI 0.023–3.75) *p* = 0.037
Endometrium [Narkiewicz] [39]	*N* = 36 all females, age not reported	*N* = 88 all females, age not reported	✓		✓	✓	✓ B-ACTIN		Difference between cancer and healthy control tissue *p* < 0.0001
Endometrium [Bowden] [40]	*N* = 15 all females, age not reported	*N* = 4 all females, age not reported	✓			✓	✓ GADPH		HtrA1 mRNA significantly lower in tumor than in normal endometrium (*p* < 0.0001)
Pleura [15]	*N* = 70 F = 29, M = 41 median age, 65 range, 45–81	–		✓	✓			✓	HtrA1 (+): HR 1 (reference category) HtrA1 (++): HR 0.65 (95% CI 0.348–0.876) HtrA1 (+++): HR 0.26 (95% CI 0.122–0.454) *p* < 0.00
Ovary [41]	*N* = 44 all females, age not reported	*N* = 19 all females, age not reported	✓		✓	✓	✓ B-ACTIN		HtrA1 mRNA significantly decreased in tumor compared with normal ovarian tissue (*p* < 0.0001) but not for HtrA1 protein (*p* = 0.452)

*M* Males, *F* Females, (*y*) years, *sd* standard deviation, *OS* overall survival

^a^The hazard ratio (HR) and 95% Confidence Interval (CI) were used as a measure of effect size. When the HR and 95% CI were not reported in the publications, they were estimated from the Kaplan-Meier curves according to Parmar et al. [28], Tierney et al. [29], and Williamson et al. [30]

### Statistical analysis

For the number of studies to be included in the meta-analysis, we made reference to Davey J et al. [21].

Odds Ratios (ORs) [22, 23], with 95% confidence interval (CI) and *p* value, were used as a measure of effect size when comparing C to HC tissue and C to NL tissue. The scores of immunostaining of HtrA1 expression reported in each study, were ranked as follow: samples ranked as 0, 1 or negative, were classified as “low expression” and those ranked as 2, 3 or 4, were classified as “medium-high expression”.

Effect sizes were pooled across studies to obtain an overall effect size. A random-effects model was applied as a conservative approach to account for different sources of variation among studies. Heterogeneity across studies was assessed using Q statistic, I2, Tau, and Tau2. A significant Q value indicated the absence of homogeneity of results among studies.

In addition, to complete the explanation of heterogeneity across study results, moderator analyses were conducted if there were at least 5 studies. The moderators evaluated by meta-regressions were sample size magnitude, % of female, mean age of both genders as appropriated, and year of publication.

Sensitivity analysis was conducted to check the stability of study findings and estimate how the overall effect size would be modified by removal of one study.

Publication bias analyses were performed when there were at least 4 studies, to control for the fact that published studies may have a larger mean effect size than unpublished studies [24]. The funnel plot, namely a scatter plot of the effect sizes estimated from individual studies against a measure of their precision (i.e. their standard error), was examined; in absence of bias, its shape should be a symmetric inverted funnel. Egger’s regression method [25] was applied to test funnel plot asymmetry. When the results of this analysis are non-significant, there is no publication bias. Finally, the trim and fill procedure was used to evaluate the effect of potential data censoring on meta-analysis results [26]. In this approach, the absence of publication bias is indicated by zero trimmed studies, or if trimmed studies are present, by trivial differences between observed and estimated effect sizes [27].

Prognostic studies were analyzed using the hazard ratio (HR) and its 95% CI as a measure of effect size. To assess the effect of HtrA1 expression on overall survival, patients were dichotomized into two classes: medium-high and low HtrA1 expression. When the HR and 95% CI were not reported in the papers, they were estimated from Kaplan-Meier curves according to Parmar et al. [28], Tierney et al. [29], and Williamson et al. [30]. Statistical analysis was performed using Prometa 3.

## Results

Search of the electronic databases according to the above-listed criteria found 59 papers, whereas the manual search found none. There were no duplicates (Additional file 1: Figure S1). In the first phase, 40 papers were excluded because they did not match the inclusion criteria. In the second phase, 19 full papers were examined and 4 more were excluded because they did not match the outcomes of interest [14, 31–33]. Eventually, 15 papers assessing HtrA1 expression at 10 tumours sites (stomach, liver, bladder, breast, esophagus, thyroid, endometrium, pleura, ovary, colorectum cancer) were included in the meta-analysis [9–13, 15, 17, 34–41].

In each meta-analysis, sensitivity analyses indicated stability of meta-analytic findings.

### C versus HC tissue: Expression of HtrA1 in protein and mRNA

**Fig. 1:**
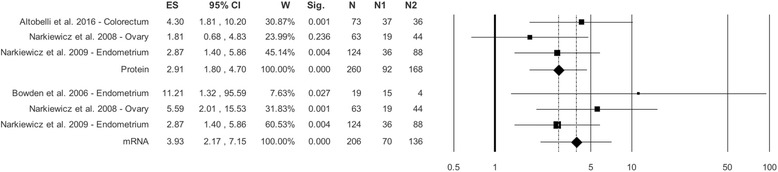
Forest plot. HtrA1 protein and mRNA in Cancer Tissue versus Healthy Control Tissue. LEGEND: ES=OR

There were 3 studies of HtrA1 expression as protein [13, 39, 41] and 3 of HtrA1 expression as mRNA [39–41]. As regards HtrA1 expression as protein the overall effect size in C vs HC was OR = 2.91 (1.80–4.70), *p* < 0.0001; Q = 1.68, I^2^ = 0.00, *p* = 0.432 (Fig. 1, Table 2). As regards HtrA1 expression as mRNA overall effect size in C vs HC was OR = 3.93 (2.17–7.15), p < 0.0001; Q = 2.12, I2 = 5.81, *P* = 0.346 (Fig. 1, Table 2).

**Table 2:** Results of the Meta-analysis

	POOLED ANALYSIS		HETEROGENEITY					PUBLICATION BIAS				
	*ES (CI)*	*P-value*	*Q*	*I* ^*2*^	*P-value*	*T* ^*2*^	*T*	*Egger’s*		*Begg and Mazdumdar’s*		
								*T*	*P-value*	*Z*	*P-value*	
Cancer vs Healthy Controls												
Protein (k = 3) [13,41,39]	2.91 (1.80;4.70)	< 0.0001	1.68	0.00	0.432	0.00	0.00	–	–	–	–	Fig. 1
mRNA (k = 3) [39–41]	3.93 (2.17;7.15)	< 0.0001	2.12	5.81	0.346	0.02	0.14	–	–	–	–	Fig. 1
Cancer vs Normal-Looking Tissue												
Protein (k = 6) [9,12,13,17,35,36]	2.93 (2.21;3.90)	< 0.0001	4.42	0.00	0.491	0.00	0.00	−1.10	0.334	−1.32	0.188	Fig. 2a, b
mRNA (k = 5) [12,17,34–36]	2. 80 (1.81;4.32)	< 0.0001	9.01	55.59	0.061	0.14	0.37	0.28	0.798	0.00	1.000	Fig. 2a, c
SURVIVAL												
Protein (k = 5) [10,15,35,36,38]	0.51 (0.37;0.70)	0.005	2.29	0.00	0.682	0.00	0.00	0.14	0.897	0.49	0.624	Fig. 3a, b
mRNA (k = 2) [11,36]	0.57 (0.31;1.06)	0.076	1.21	17.03	0.272	0.04	0.19	–	–	–	–	Fig. 3a

### C versus NL tissue: Expression of HtrA1 protein

**Fig. 2:**
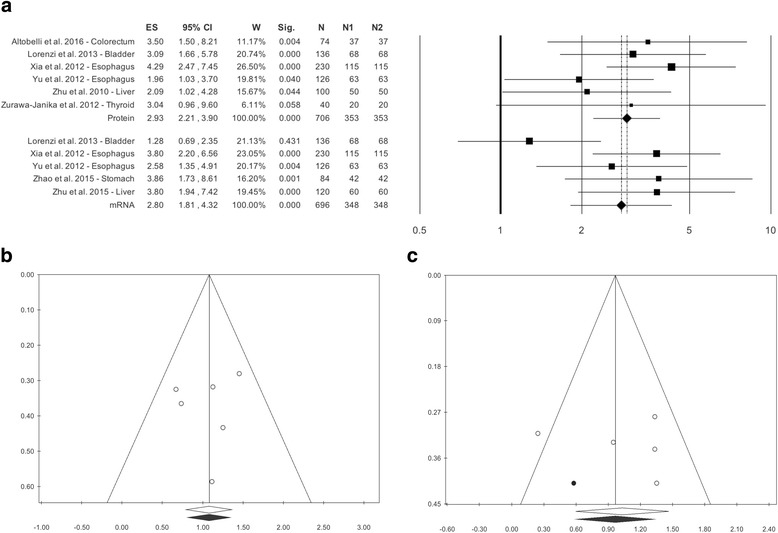
**a** Forest plot. HtrA1 protein and mRNA in Cancer Tissue versus Normal-Looking Tissue **b** Funnel plot. HtrA1 protein in Cancer Tissue versus Normal-Looking Tissue. **c** Funnel plot. HtrA1 mRNA in Cancer Tissue versus Normal-Looking Tissue

As regards C vs NL tissue, there were 6 studies [9, 12, 13, 17, 35, 36]. The overall effect size was OR = 2.93 (2.21–3.90), p < 0.0001, with Q = 4.42, I^2^ = 0.00, *p* = 0.491 (Table 2, Fig. 2a).

Publication bias analysis did not highlight differences between observed and estimated values (0 trimmed studies), and Egger’s linear test was not statistically significant: *p* = 0.334 (Table 2, Fig. 2b). Meta-regressions for sample size magnitude, % of females, mean age of both gender, and year of publication did not show statistically significant differences: beta = 0.01, *p* = 0.177; beta = 0.02, *p* = 0.083, beta = 0.00, *p* = 0.919; beta = 0.08, *p* = 0.422 (Additional file 5: Figure S2A, B, C, D).

### C versus NL tissue: Expression of HtrA1 mRNA

As regards C vs NL tissue, there were 5 studies [12, 17, 34–36]. The overall effect size was OR = 2.80 (1.81–4.32), *p* < 0.0001, Q = 9.01, *p* = 0.061, I^2^ = 55.59, without statistically significant heterogeneity (Table 2, Fig. 2a).

Although publication bias analysis trimmed 1 study the result of Egger’s linear test, *p* = 0.798, was not statistically significant (Fig. 2c). Meta-regressions for sample size magnitude, % of females, and year of publication did not disclose statistically significant differences: beta = 0.00, *p* = 0.816; beta = 0.02, *p* = 0.218; beta = 0.09, *p* = 0.633; (Additional file 6: Figure S3A, B, C). Mean age was not evaluated because too few studies reported data for both genders.

### HtrA1 expression and survival analysis

**Fig. 3:**
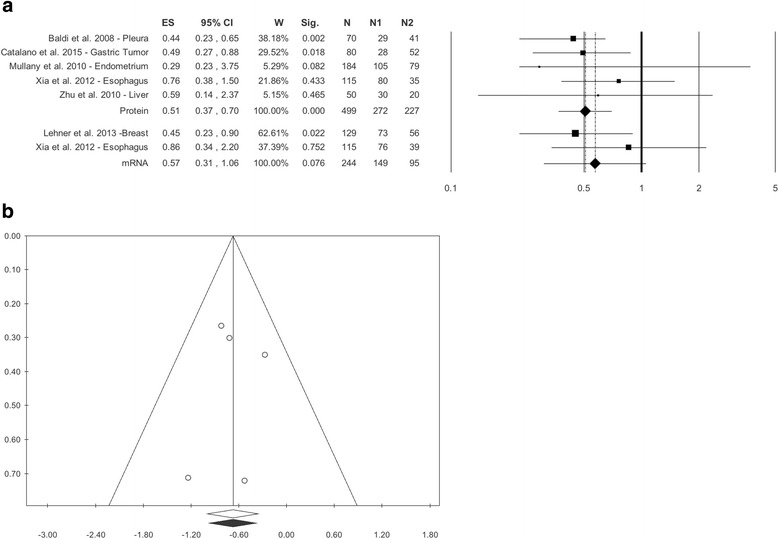
**a** Forest Plot. Survival Analysis in Protein and mRNA, **b**. Funnel Plot. Survival Analysis in Protein. LEGEND: ES=HR

The second aim of the meta-analysis was to assess overall survival in relation to low and medium-high HtrA1 expression in cancers. The literature search retrieved 7 studies: 2 investigating HtrA1 as mRNA [11, 36] and 5 assessing HtrA1 as protein [10, 15, 35, 36, 38]. As regards mRNA, the overall effect size was HR = 0.57 (0.31–1.06), *p* = 0.076, without statistical heterogeneity, Q = 1.21, *p* = 0.272, I^2^ = 17.03 (Table 2, Fig. 3a). As regards HtrA1 measured as protein, the overall effect size was HR = 0.51 (0.37–0.70), *p* < 0.0005, without significant heterogeneity, Q = 2.29, *p* = 0.682, I^2^ = 0.00 (Table 2, Fig. 3a).

Publication bias analysis and meta-regression were performed only for the studies assessing HtrA1 as protein. The funnel plot did not show differences between observed and estimated values (0 trimmed studies), and Egger’s linear test was not statistically significant, *p* = 0.897 (Table 2, Fig. 3b). Meta-regressions for sample size magnitude, % of females and year of publication did not show statistical differences: beta = 0.00, *p* = 0.806; beta = − 0.00, *p* = 0.741; beta = 0.03, *p* = 0.598 (Additional file 7: Figure S4A, B, C, D).

## Discussion

In recent years, mounting biological knowledge of disease processes and advances in molecular technologies have increased the interest in biomarkers. In fact, biomarkers are used for patient assessment in multiple clinical settings, both to distinguish an individual with disease from one without disease and to discriminate one disease from another, i.e. for differential diagnosis. Ad hoc immune-histochemical markers can also be used to try and identify the tissue where a tumor has had its origin, to assess the risk of relapse, as well as to evaluate prognosis or response to therapy.

In the present study, a meta-analysis was conducted to assess the potential role of HtrA1 as a tumor marker and/or prognostic factor in a number of tumors. Most current studies focus on the role of HtrA1 in tumor development and progression through analysis of its expression as mRNA [11, 12, 17, 34–36, 39–41] or protein [9, 10, 13, 15, 17, 35–38], probably because proteins are more varied than DNA or RNA and therefore carry more information than nucleic acids. Indeed, alternative splicing and more than 100 unique post-translational modifications result in tens (and possibly hundreds) of protein species from each gene. Moreover, a number of physiological changes are mediated post-transcriptionally and are not revealed at the nucleic acid level. Proteins are also more dynamic and reflective of cellular physiology. For instance, the occurrence of a single double-strand DNA break in a cell is rapidly amplified into a protein phosphorylation cascade. Thus, protein-based markers provide a fine, specific, and accurate representation of the condition being investigated.

Based on these considerations, in this work the studies assessing HtrA1 expression as mRNA and protein were evaluated separately. An important finding of the study was that, in the absence of significant statistical heterogeneity, there was a statistically significant difference between the tumor samples analyzed and HC and NL tissue specimens; this applied both to HtrA1 measured as mRNA and as protein (Table 2, Figs. 1 and 2a,). The results regarding the non-heterogeneity of studies were supported by the analysis of the moderators used, i.e. sample size magnitude, % of females, mean age for both genders, and year of publication, which did not exhibit statistically significant differences (Additional file 5: Figure S2A, D). Publication bias was highlighted neither by the funnel plot nor by Egger’s or Begg’s test. A consistent finding of this work, that was described in all the studies that tested this aspect [9–13, 15, 17, 34–41], was that HtrA1 levels are higher in HC or NL tissue than in diseased tissue from patients with a variety of tumors.

The meta-analysis of the prognostic studies showed, in the absence of significant heterogeneity among the studies examined, a different survival according to HtrA1 expression. These findings are in line with the literature, since in some studies HtrA1 has been found to modulated cisplatin- and paclitaxel-induced cytotoxicity and low HtrA1 levels have been seen to correlate with a poor response to drug treatment, whereas higher levels correlated with a greater response [38, 42].

Baldi et al. [15] have demonstrated a relationship between HtrA1 expression level and survival in patients with malignant mesothelioma, suggesting that HtrA1 expression can be used as a prognostic parameter for this tumor type. Analysis of HtrA1 levels in relation to overall survival and disease free survival in breast cancer [11] indicated that patients with higher HtrA1 levels had a better prognosis.

Analysis of the moderators assessed in the works selected found no statistically significant differences and there was no publication bias (Additional file 7: Figure S4).

However, some weaknesses in the data suggest that the results of the present meta-analysis should be taken with caution. First of all, most of the studies are descriptive, preventing a causal inference between reduced HtrA1 levels and cancer. Secondly, the small number of works on the same tumor prevents an analysis by tumor in relation to individual organs. Thirdly, since parameters such as histological type and tumor grade and stage have not been addressed in all the studies, it is impossible to establish how HtrA1 expression varies in relation to these factors. Finally, the different HtrA1 expression, found in papers assessing the same cancer type, could correlate to histological grading, metastasis, and degree of cell differentiation, but were not related to patients’ age or gender, as demonstrated in hepatocellular carcinoma [12], esophageal carcinoma [35, 36] and endometrial cancer [10, 40, 41]. This means that the reduced HtrA1 expression may be closely associated to tumor development. The lack of difference in HtrA1 expression reported by Catalano et al. (2011 [38]) may be due to heterogeneous baseline characteristics of their patients, like constitutional genetic factors and other variables, which as the authors themselves stated were not investigated.

## Conclusions

The present data may provide a contribution to future research work directed at exploring the role of HtrA1 in tumor development and progression and at establishing whether it may become a promising tissue marker for some tumors. Finally, the present work suggests that clinical investigations sharing a similar approach, especially in terms of study design, should be carried out to improve comparability across studies.

## Additional files

Additional file 1: Figure S1. Flow-chart of research strategy. (PDF 252 kb)

Additional file 2: Table S1. Excluded papers. (PDF 288 kb)

Additional file 3: Table S2. PRISMA Check-list. (PDF 267 kb)

Additional file 4: Table S3. Newcastle-Ottawa evaluation for case-control studies. (PDF 82 kb)

Additional file 5: Figure S2. Normal-Looking Tissue. Meta-regression of HtrA1 protein: A. Sample size, B. % Female, C. Mean age of entire sample, D. Publication year. (PDF 218 kb)

Additional file 6: Figure S3. Normal-Looking Tissue. Meta-regression of HtrA1 mRNA: A. Sample size, B. % Female, C. Mean age of entire sample, D. Publication year. (PDF 1270 kb)

Additional file 7: Figure S4. Survival studies. Meta-regression of HtrA1 protein: A. Sample size, B. % Female, C. Mean age of entire sample, D. Publication year (PDF 149 kb)

## Abbreviations

C: cancer tissue
HC: healthy control
HR: hazard ratio; OR: odds ratio
NL: normal-looking
