# Supplementary figures and images for: HtrA1 as a promising tissue marker in cancer: a meta-analysis
Source: BMC Cancer. 2018 Feb 6;18:143. doi: 10.1186/s12885-018-4041-2 (PMC5801749; doi:10.1186/s12885-018-4041-2)

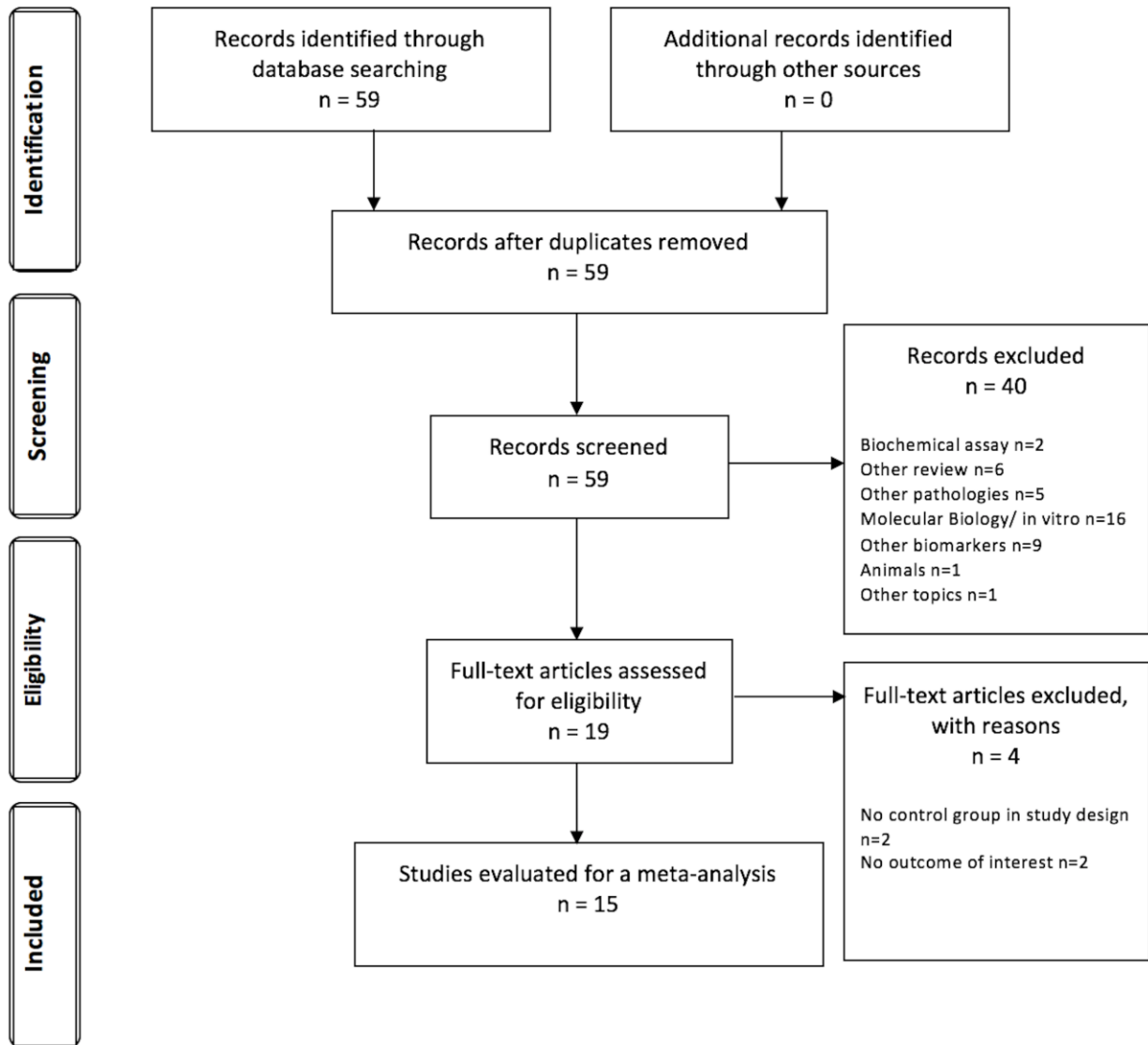

Supplement: Supplementary file 1 — Flow-chart of research strategy. (PDF 252 kb) [file 12885_2018_4041_MOESM1_ESM.pdf]
