# Supplementary material for: HtrA1 as a promising tissue marker in cancer: a meta-analysis
Source: BMC Cancer. 2018 Feb 6;18:143. doi: 10.1186/s12885-018-4041-2 (PMC5801749; doi:10.1186/s12885-018-4041-2)
Supplement: Supplementary file 2 — Excluded papers. (PDF 288 kb) [file 12885_2018_4041_MOESM2_ESM.pdf]

Additional file 2: Table S1. Excluded studies

Reference no#	Authors, title, journal
1	Ciferri C, Lipari MT, Liang WC, Estevez A, Hang J, Stawicki S, Wu Y, Moran P, Elliott M, Eigenbrot C, Katschke KJ, van Lookeren Campagne M, Kirchhofer D. The trimeric serine protease HtrA1 forms a cage-like inhibition complex with an anti-HtrA1 antibody. Biochem J. 2015 Dec 1;472(2):169-81
2	Friedrich U, Datta S, Schubert T, Plössl K, Schneider M, Grassmann F, Fuchshofer R, Tiefenbach KJ, Längst G, Weber BH. Synonymous variants in HTRA1 implicated in AMD susceptibility impair its capacity to regulate TGF-β signaling. Hum Mol Genet. 2015 Nov 15;24(22):6361-73
3	Altobelli E, Marzioni D, Lattanzi A, Angeletti PM. HtrA1: Its future potential as a novel biomarker for cancer. Oncol Rep. 2015 Aug;34(2):555-66.
4	Minchenko DO, Kharkova AP, Karbovskyi LL, Minchenko OH. Expression of insulin-like growth factor binding protein genes and its hypoxic regulation in U87 glioma cells depends on ERN1 mediated signaling pathway of endoplasmic reticulum stress. Endocr Regul. 2015 Apr;49(2):73-83.
5	Bao W, Zhu F, Duan Y, Yang Y, Cai H. HtrA1 resensitizes multidrug-resistant hepatocellular carcinoma cells by targeting XIAP. Biomed Pharmacother. 2015 Mar;70:97-102.
6	Huang L, Meng Q, Zhang C, Sun Y, Bai Y, Li S, Deng X, Wang B, Yu W, Zhao M, Li X. Gene-gene interaction of CFH, ARMS2, and ARMS2/HTRA1 on the risk of neovascular age-related macular degeneration and polypoidal choroidal vasculopathy in Chinese population. Eye (Lond). 2015 May;29(5):691-8.
7	Önder E, Arıkök AT, Seçkin H, Alper M Decrease in serine protease HtrA1 expression correlates with grade and recurrence in meningiomas. Adv Med Sci. 2015 Mar;60(1):139-43.
8	Franco R, Collina F, Di Bonito M, Botti G, Montanaro D, Di Maio L, Vincenzi B, Landi G, D'Aiuto M, Caraglia M, Baldi A. HtrA1 loss is related to aggressive behavior parameters in sentinel node positive breast cancer. Histol Histopathol. 2015
9	Lorenzi T, Niţulescu EA, Zizzi A, Lorenzi M, Paolinelli F, Aspriello SD, Baniţă M, Crăiţoiu S, Goteri G, Barbatelli G, Lombardi T, Di Felice R, Marzioni D, Rubini C, Castellucci M. The novel role of HtrA1 in gingivitis, chronic and aggressive periodontitis. PLoS One. 2014 Jun 30;9(6):e96978
10	Hou Y, Lin H, Zhu L, Liu Z, Hu F, Shi J, Yang T, Shi X, Guo H, Tan X, Zhang L, Wang Q, Li Z, Zhao Y. The inhibitory effect of IFN-γ on protease HTRA1 expression in rheumatoid arthritis. J Immunol. 2014 Jul 1;193(1):130-8
11	Zielonka S, Weber N, Becker S, Doerner A, Christmann A, Christmann C, Uth C, Fritz J, Schäfer E, Steinmann B, Empting M, Ockelmann P, Lierz M, Kolmar H. Shark Attack: high affinity binding proteins derived from shark vNAR domains by stepwise in vitro affinity maturation.J Biotechnol. 2014 Dec 10;191:236-45.
12	D'Angelo V, Pecoraro G, Indolfi P, Iannotta A, D'Onofrio V, Errico ME, Indolfi C, Ramaglia M, Lombardi A, Di Martino M, Gigantino V, Baldi A, Caraglia M, De Luca A, Casale F Expression and localization of serine protease Htra1 in neuroblastoma: correlation with cellular differentiation grade. J Neurooncol. 2014 Apr;117(2):287-94.
13	Xu Y, Jiang Z, Zhang Z, Sun N, Zhang M, Xie J, Li T, Hou Y, Wu D HtrA1 downregulation induces cisplatin resistance in lung adenocarcinoma by promoting cancer stem cell-like properties. J Cell Biochem. 2014 Jun;115(6):1112-21
14	Yin W, Wang P, Wang X, Song W, Cui X, Yu H, Zhu W. Identification of microRNAs and mRNAs associated with multidrug resistance of human laryngeal cancer Hep-2 cells. Braz J Med Biol Res. 2013 Jun;46(6):546-54
15	Wang N, Eckert KA, Zomorrodi AR, Xin P, Pan W, Shearer DA, Weisz J, Maranus CD, Clawson GA. Down-regulation of HtrA1 activates the epithelial-mesenchymal transition and ATM DNA damage response pathways. PLoS One. 2012;7(6):e39446.

16	Wehr AY, Hwang WT, Blair IA, Yu KH. Relative quantification of serum proteins from pancreatic ductal adenocarcinoma patients by stable isotope dilution liquid chromatography-mass spectrometry. J Proteome Res. 2012 Mar 2;11(3):1749-58.
17	Singh N, Kuppili RR, Bose K. The structural basis of mode of activation and functional diversity: a case study with HtrA family of serine proteases. Arch Biochem Biophys. 2011 Dec 15;516(2):85-96
18	Neuhausen SL, Brummel S, Ding YC, Steele L, Nathanson KL, Domchek S, Rebbeck TR, Singer CF, Pfeiler G, Lynch HT, Garber JE, Couch F, Weitzel JN, Godwin A, Narod SA, Ganz PA, Daly MB, Isaacs C, Olopade OI, Tomlinson GE, Rubinstein WS, Tung N, Blum JL, Gillen DL. Genetic variation in IGF2 and HTRA1 and breast cancer risk among BRCA1 and BRCA2 carriers. Cancer Epidemiol Biomarkers Prev. 2011 Aug;20(8):1690-702
19	Safarinejad MR, Shafiei N, Safarinejad S. Relationship of insulin-like growth factor (IGF) binding protein-3 (IGFBP-3) gene polymorphism with the susceptibility to development of prostate cancer and influence on serum levels of IGF-I, and IGFBP-3. Growth Horm IGF Res. 2011 Jun;21(3):146-54.
20	He X, Khurana A, Maguire JL, Chien J, Shridhar V. HtrA1 sensitizes ovarian cancer cells to cisplatin-induced cytotoxicity by targeting XIAP for degradation. Int J Cancer. 2012 Mar 1;130(5):1029-35
21	Campioni M, Severino A, Manente L, Tuduce IL, Toldo S, Caraglia M, Crispi S, Ehrmann M, He X, Maguire J, De Falco M, De Luca A, Shridhar V, Baldi A The serine protease HtrA1 specifically interacts and degrades the tuberous sclerosis complex 2 protein. Mol Cancer Res. 2010 Sep;8(9):1248-60.
22	Zurawa-Janicka D, Skorko-Glonek J, Lipinska B. HtrA proteins as targets in therapy of cancer and other diseases. Expert Opin Ther Targets. 2010 Jul;14(7):665-79.
23	He X, Ota T, Liu P, Su C, Chien J, Shridhar V. Downregulation of HtrA1 promotes resistance to anoikis and peritoneal dissemination of ovarian cancer cells. Cancer Res. 2010 Apr 15;70(8):3109-18
24	Marzioni D, Quaranta A, Lorenzi T, Morroni M, Crescimanno C, De Nictolis M, Toti P, Muzzonigro G, Baldi A, De Luca A, Castellucci M. Expression pattern alterations of the serine protease HtrA1 in normal human placental tissues and in gestational trophoblastic diseases. Histol Histopathol. 2009 Oct;24(10):1213-22.
25	Chien J, Ota T, Aletti G, Shridhar R, Boccellino M, Quagliuolo L, Baldi A, Shridhar V. Serine protease HtrA1 associates with microtubules and inhibits cell migration. Mol Cell Biol. 2009 Aug;29(15):4177-87.
26	Chien J, He X, Shridhar V. Identification of tubulins as substrates of serine protease HtrA1 by mixture-based oriented peptide library screening. J Cell Biochem. 2009 May 15;107(2):253-63.
27	Clawson GA, Bui V, Xin P, Wang N, Pan W. Intracellular localization of the tumor suppressor HtrA1/Prss11 and its association with HPV16 E6 and E7 proteins. J Cell Biochem. 2008 Sep 1;105(1):81-8.
28	Ryan CJ, Harzstark AH, Rosenberg J, Lin A, Claros C, Goldfine ID, Kerner JF, Small EJ, Youngren JF. A pilot dose-escalation study of the effects of nordihydroguareacetic acid on hormone and prostate specific antigen levels in patients with relapsed prostate cancer. BJU Int. 2008 Feb;101(4):436-9. doi: 10.1111/j.1464-410X.2007.07330.x. Erratum in: BJU Int. 2008 Jun;101(11):1483. Youngren, Jack F [added].
29	Zurawa-Janicka D, Kobiela J, Stefaniak T, Wozniak A, Narkiewicz J, Wozniak M, Limon J, Lipinska B. Changes in expression of serine proteases HtrA1 and HtrA2 during estrogen-induced oxidative stress and nephrocarcinogenesis in male Syrian hamster. Acta Biochim Pol. 2008;55(1):9-19.
30	Bensalah K, Lotan Y, Karam JA, Shariat SF. New circulating biomarkers for prostate cancer. Prostate Cancer Prostatic Dis. 2008;11(2):112-20
31	Canfield AE, Hadfield KD, Rock CF, Wylie EC, Wilkinson FL. HtrA1: a novel regulator of physiological and pathological matrix

	mineralization? Biochem Soc Trans. 2007 Aug;35(Pt 4):669-71
32	Sano A, Sangai T, Maeda H, Nakamura M, Hasebe T, Ochiai A. Kallikrein 11 expressed in human breast cancer cells releases insulin-like growth factor through degradation of IGFBP-3. Int J Oncol. 2007 Jun;30(6):1493-8.
33	Oh SH, Lee OH, Schroeder CP, Oh YW, Ke S, Cha HJ, Park RW, Onn A, Herbst RS, Li C, Lee HY. Antimetastatic activity of insulin-like growth factor binding protein-3 in lung cancer is mediated by insulin-like growth factor-independent urokinase-type plasminogen activator inhibition. Mol Cancer Ther. 2006 Nov;5(11):2685-95.
34	Esposito V, Campioni M, De Luca A, Spugnini EP, Baldi F, Cassandro R, Mancini A, Vincenzi B, Groeger A, Caputi M, Baldi A. Analysis of HtrA1 serine protease expression in human lung cancer. Anticancer Res. 2006 Sep-Oct;26(5A):3455-9.
35	Severi G, Morris HA, MacInnis RJ, English DR, Tilley WD, Hopper JL, Boyle P, Giles GG. Circulating insulin-like growth factor-I and binding protein-3 and risk of prostate cancer. Cancer Epidemiol Biomarkers Prev. 2006 Jun;15(6):1137-41.
36	Chien J, Aletti G, Baldi A, Catalano V, Muretto P, Keeney GL, Kalli KR, Staub J, Ehrmann M, Cliby WA, Lee YK, Bible KC, Hartmann LC, Kaufmann SH, Shridhar V. Serine protease HtrA1 modulates chemotherapy-induced cytotoxicity. J Clin Invest. 2006 Jul;116(7):1994-2004.
37	Liao Y, Abel U, Grobholz R, Hermani A, Trojan L, Angel P, Mayer D. Up-regulation of insulin-like growth factor axis components in human primary prostate cancer correlates with tumor grade. Hum Pathol. 2005 Nov;36(11):1186-96
38	Grau S, Baldi A, Bussani R, Tian X, Stefanescu R, Przybylski M, Richards P, Jones SA, Shridhar V, Clausen T, Ehrmann M Implications of the serine protease HtrA1 in amyloid precursor protein processing. Proc Natl Acad Sci U S A. 2005 Apr 26;102(17):6021-6.
39	Inman BA, Harel F, Audet JF, Meyer F, Douville P, Fradet Y, Lacombe L. Insulin-like growth factor binding protein 2: an androgen-dependent predictor of prostate cancer survival. Eur Urol. 2005 May;47(5):695-702.
40	Serrano D, Baglietto L, Johansson H, Mariette F, Torrisi R, Onetto M, Paganuzzi M, Decensi A. Effect of the synthetic retinoid fenretinide on circulating free prostate-specific antigen, insulin-like growth factor-I, and insulin-like growth factor binding protein-3 levels in men with superficial bladder cancer. Clin Cancer Res. 2005 Mar 1;11(5):2083-8.
41	Chien J, Staub J, Hu SI, Erickson-Johnson MR, Couch FJ, Smith DI, Crowl RM, Kaufmann SH, Shridhar V. A candidate tumor suppressor HtrA1 is downregulated in ovarian cancer. Oncogene. 2004 Feb 26;23(8):1636-44.
42	Nam RK, Zhang WW, Trachtenberg J, Jewett MA, Emami M, Vesprini D, Chu W, Ho M, Sweet J, Evans A, Toi A, Pollak M, Narod SA. Comprehensive assessment of candidate genes and serological markers for the detection of prostate cancer. Cancer Epidemiol Biomarkers Prev. 2003 Dec;12(12):1429-37.
43	Harman SM, Metter EJ, Blackman MR, Landis PK, Carter HB; Baltimore Longitudinal Study on Aging. Serum levels of insulin-like growth factor I (IGF-I), IGF-II, IGF-binding protein-3, and prostate-specific antigen as predictors of clinical prostate cancer. J Clin Endocrinol Metab. 2000 Nov;85(11):4258-65.
44	Zumbrunn J, Trueb B. Primary structure of a putative serine protease specific for IGF-binding proteins. FEBS Lett. 1996 Dec 2;398(2-3):187-92.
