# Supplementary material for: HtrA1 as a promising tissue marker in cancer: a meta-analysis
Source: BMC Cancer. 2018 Feb 6;18:143. doi: 10.1186/s12885-018-4041-2 (PMC5801749; doi:10.1186/s12885-018-4041-2)
Supplement: Supplementary file 4 — Newcastle-Ottawa evaluation for case-control studies. (PDF 82 kb) [file 12885_2018_4041_MOESM4_ESM.pdf]

Supplementary Table 3: New Castle-Ottawa scale assesment for case-control studies

**Study ID**	**Selection**				**Comparability**	**Exposure**	
	Is the case definition adequate	Representativeness of the cases	Selection of Controls	Definition of Controls	Comparability of cases and controls on the basis of the design or analysis	Ascertainment of exposure	Same method of ascertainment for cases and controls
**Zhu (Liver, mRNA)**	***A**	***B**	**B**	***A**	***B**	***A**	***A**
**Zhao**	***A**	***A**	**B**	***A**	***B**	***A**	***A**
**Lorenzi**	***A**	***A**	**B**	***A**	***B**	***A**	***A**
**Lehner**	***A**	***A**	**B**	***A**	***B**	***A**	***A**
**Yu**	***A**	***A**	**B**	***A**	***B**	***A**	***A**
**Xia**	***A**	***A**	**B**	***A**	***B**	***A**	***A**
**Zhu (Liver, protein)**	***A**	***B**	**B**	***A**	***B**	***A**	***A**
**Catalano**	***A**	***A**	**B**	***A**	***B**	***A**	***A**
**Zurawa-Janika**	***A**	***B**	**B**	***A**	***B**	***A**	***A**
**Mullany**	***A**	***A**	**B**	***A**	***B**	***A**	***A**
**Narkiewicx (endometrium)**	***A**	***A**	**B**	***A**	***B**	***A**	***A**
**Bowden**	***A**	***A**	**B**	***A**	***B**	***A**	***A**
**Baldi**	***A**	***A**	**B**	***A**	***B**	***A**	***A**
**Narkiewicx**	***A**	***A**	**B**	***A**	***B**	***A**	***A**

(ovary)

Selection

1) Is the case definition adequate? a) yes, with independent validation ✵ b) yes, eg record linkage or based on self reports c) no description
2) Representativeness of the cases a) consecutive or obviously representative series of cases ✵ b) potential for selection biases or not stated
3) Selection of Controls a) community controls ✵ b) hospital controls c) no description
4) Definition of Controls a) no history of disease (endpoint) ✵b) no description of source

Comparability

1) Comparability of cases and controls on the basis of the design or analysis
 a) study controls for age and sex ✵
 b) study controls for any additional factor ✵

Exposure

1) Ascertainment of exposure a) secure record (eg surgical records) ✵ b) structured interview where blind to case/control status ✵
 c) interview not blinded to case/control status d) written self report or medical record only e) no description
2) Same method of ascertainment for cases and controls a) yes ✵ b) no
