# Supplementary material for: HtrA1 as a promising tissue marker in cancer: a meta-analysis
Source: BMC Cancer. 2018 Feb 6;18:143. doi: 10.1186/s12885-018-4041-2 (PMC5801749; doi:10.1186/s12885-018-4041-2)
Supplement: Supplementary file 7 — Survival studies. Meta-regression of HtrA1 protein: A. Sample size, B. % Female, C. Mean age of entire sample, D. Publication year (PDF 149 kb) [file 12885_2018_4041_MOESM7_ESM.pdf]

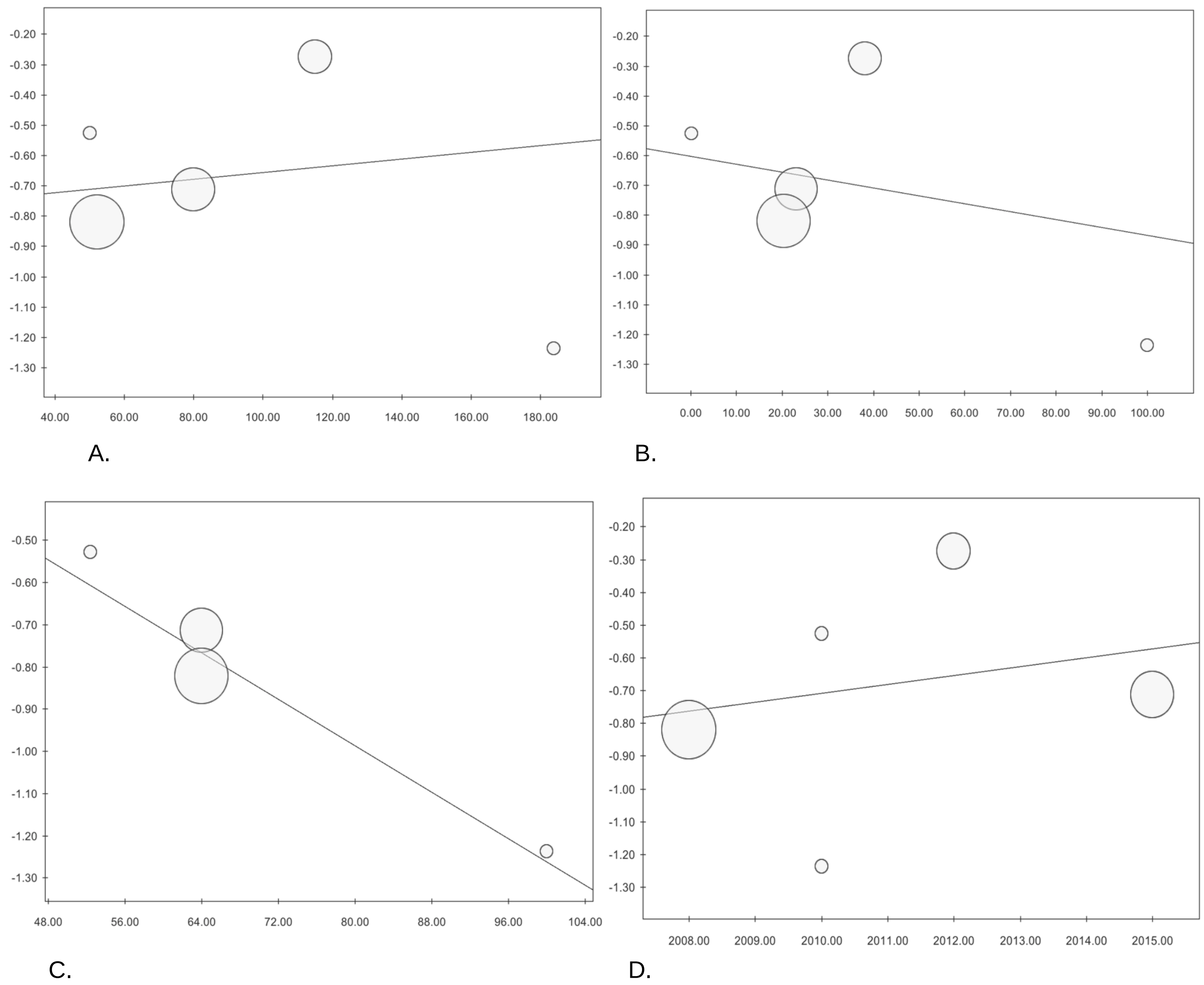

FIGURE S4. Survival studies. Meta-regression of HtrA1 protein: A. Sample size, B. % Female, C. Mean age of entire sample, D. Publication year
